# Impact of the coxsackievirus and adenovirus receptor on the adenoma–carcinoma sequence of colon cancer

**DOI:** 10.1038/bjc.2011.116

**Published:** 2011-04-05

**Authors:** K Stecker, M Vieth, A Koschel, B Wiedenmann, C Röcken, M Anders

**Affiliations:** 1 Department of Internal Medicine, Divisions of Gastroenterology and Hepatology, Charité Medical School, Campus Virchow, Augustenburgerplatz 1, Berlin 13353, Germany; 2 Institute of Pathology, Klinikum Bayreuth, Preuschwitzer Str. 101, Bayreuth 95445, Germany; 3 Institute of Pathology, Christian-Albrechts-University, Kiel, Arnold-Heller-Str. 3, Kiel 24105, Germany; 4 Department of Interdisciplinary Endoscopy, University Hospital Hamburg Eppendorf, Martinistr. 52, Hamburg 20246, Germany

**Keywords:** coxsackie adenovirus receptor, adenoma–carcinoma sequence, colon cancer

## Abstract

**Background:**

Coxsackie and adenovirus receptor (CAR) has been suggested to function as a tumour suppressor. Its impact on the adenoma–carcinoma sequence of the colon, however, is unclear.

**Methods:**

Coxsackie and adenovirus receptor was analysed in non-cancerous and neoplastic colon samples using immunohistochemistry and quantitative RT–PCR. The function of CAR in colon cancer cell lines was determined following application of CAR siRNA or ectopic expression of a human full-length CAR cDNA.

**Results:**

Compared with healthy mucosa, increased CAR-mRNA expression was found in adenomas, whereas primary cancers and metastases displayed a marked decline. At the plasma membrane, CAR was present in normal mucosa samples (93%), adenomas, and metastases (100% ea.), whereas in colon cancers, it was found less frequently (49%, *P*<0.0001). Cytoplasmic CAR immunopositivity increased from normal mucosa (22%), to adenomas (73%, *P*=0.0006), primary cancers (83%, *P*<0.0001), and metastases (67%, *P*=0.0019). In cancer cell lines, CAR inhibition resulted in increased proliferation, whereas enforced ectopic CAR expression led to opposite results. Blocking the extracellular portion of CAR increased cell invasion *in vitro*. In mice, xenotransplants of colon cancer cells with enforced CAR expression formed significantly smaller tumours, whereas CAR inhibition increased the formation of liver metastases.

**Conclusion:**

We conclude that CAR facilitates complex effects during colon carcinogenesis, potentially mediated by its stage-dependent subcellular distribution; high CAR expression potentially prevents apoptosis in adenomas, loss of CAR at the plasma membrane promotes growth, and dissemination of primary cancers, and high membranous CAR presence may support the establishment of distant metastases.

Colon cancer represents one of the leading cancer entities worldwide (Parkin *et al*, 2005). It most commonly develops from adenomas, referred to as ‘adenoma–carcinoma sequence’ (Cho and Vogelstein, 1992). The clinical outcome of colon cancer is critically determined by local tumour growth, as well as by the presence of local and distant metastases. For both, invasion and metastatic spread, an impaired adhesion of cancer cells is considered as a crucial prerequisite. Although previously studied, particularly for the adherens junction protein E-cadherin (Buda and Pignatelli, 2004), investigation of tight junctions (TJs) in colon cancer has turn out to be of interest in recent years. Tight junctions represent a complex of proteins in epithelial and endothelial cells separating the apical and basolateral plasma membrane (Satoh *et al*, 1996; Tsukita *et al*, 1996). They maintain cellular polarity as well as tissue integrity, and regulate the paracellular transport (Tsukita and Furuse, 2000). An impaired TJ barrier has been found in human colon adenomas and in the ‘pre-neoplastic’ colon of rats treated with the carcinogen 1, 2-dimethylhydrazine (DMH) (Soler *et al*, 1999). In colon cancer, a decreased presence of TJ proteins has been described for instance for ZO-1, occludin, and claudin 8, in part, correlating with poor cancer differentiation, disease recurrence, and an adverse prognosis (Kimura *et al*, 1997; Resnick *et al*, 2005; Grone *et al*, 2007). In contrast, TJ protein upregulation in colon cancers compared with normal colon mucosa has been noted for instance for claudins 1 and 12 (Miwa *et al*, 2000; Grone *et al*, 2007). These observations point to a complex dysregulation of TJ proteins in colon carcinogenesis. The functional role of this phenomenon, however, has rarely been investigated: Claudin 1 has been shown to induce epithelial–mesenchymal transdifferentiation in cultured human colon cancer cell lines, to promote the growth of xenograft tumours and the formation of metastases in athymic mice (Dhawan *et al*, 2005). A more detailed understanding of the function of TJs in colon cancer, however, is still missing.

The coxsackie adenovirus receptor (CAR), a transmembrane glycoprotein, was initially characterised as a viral attachment site on the surface of epithelial cells (Bergelson *et al*, 1997). Later on it was identified as a component of the TJ complex, an interacting partner for a number of TJ proteins, and a regulator of TJ formation (Cohen *et al*, 2001; Sollerbrant *et al*, 2003; Coyne *et al*, 2004; Excoffon *et al*, 2004; Mirza *et al*, 2005; Raschperger *et al*, 2006). In the context of malignant diseases, reduced CAR presence was found in several solid cancers partially associated with poor differentiation, increased infiltration, and an unfavourable clinical outcome (Rauen *et al*, 2002; Sachs *et al*, 2002; Matsumoto *et al*, 2005; Korn *et al*, 2006; Buscarini *et al*, 2007; Okegawa *et al*, 2007; Anders *et al*, 2009b). In primary colon cancers, a high variability in CAR expression was found, with approximately 75% of the cases showing CAR downregulation in (Zhang *et al*, 2008). Functionally, loss of CAR has been suggested to weaken intercellular adhesion, to increase proliferation, and to promote migration as well as invasion of cancer cells (Okegawa *et al*, 2000, 2001; Bruning and Runnebaum, 2003, 2004; Huang *et al*, 2005; Wang *et al*, 2005; Anders *et al*, 2009a, 2009b). Recently, we demonstrated that downregulation of *α*-catenin following reduced expression of CAR substantially contributes to these phenomena (Stecker *et al*, 2009). On the basis of these findings, a tumour-suppressive role of CAR in human cancers has been postulated. In contrast, it has also been speculated that CAR advances the development of adenocarcinomas, as elevated CAR expression was found in early-stage breast and oesophageal cancer (Anders *et al*, 2003b; Kuster *et al*, 2010). Moreover, it has been demonstrated that CAR protects adenocarcinoma cells against apoptosis and is needed for efficient tumour formation (Qin *et al*, 2004; Bruning *et al*, 2005).

Prompted by these findings, suggestive of a complex role of CAR in carcinogenesis, we aimed to clarify the influence of CAR on the pathobiology of colon cancers. We determined CAR presence in the course of the adenoma–carcinoma sequence and studied CARs function *in vitro* and *in vivo* using cultured colon cancer cell lines, following ectopically regulated CAR expression.

## Materials and methods

### Study population and tissues

Tissue samples were obtained from 82 patients (43 men, mean age 70 years; range: 43–91 years) comprehending normal colon mucosa (*n*=27), tubular adenomas (*n*=22), primary colon adenocarcinomas (*n*=53), and metastatic colon cancers (total *n*=22: liver *n*=6, lung *n*=4, lymph nodes *n*=12). Staging and diagnosis was assessed according to the WHO classification (Hamilton, 2000) and the TNM staging, set out by the International Union against cancer (Wittekind, 2003).

### Quantitative mRNA determination

Total RNA was isolated from three consecutive 15-*μ*m tissue sections containing at least 80% of the tissue of interest using the ABSOLUTELY RNA FFPE KIT (Stratagene, Amsterdam, The Netherlands) according to the manufacturer's protocol. Upon reverse transcription of 50 ng of total RNA with random hexamer primers using the ‘first-strand synthesis system for RT–PCR’ (Invitrogen, Karlsruhe, Germany), real-time quantification was carried out as described previously (Anders *et al*, 2003b) on a Stratagene MX3000P cycler. Quantification was assessed by the comparative Δ*C*_T_ method normalising *C*_T_ values to *β*-actin. Complementary DNA derived from Chinese hamster ovary cells (CHO, CHO-CAR; a kind gift of Dr J Bergelson, Division of Infectious Diseases, Children's Hospital of Philadelphia, Philadelphia, PA, USA) was used as negative and positive control, respectively.

### Immunohistochemistry

Immunohistochemical staining was carried out as described previously (Anders *et al*, 2009b). In brief, tissue sections were deparaffinised, rehydrated, and submitted to antigen retrieval by microwave treatment. Anti-CAR (H-300: sc-15405, Santa Cruz Biotechnology, Inc., Santa Cruz, CA, USA; 1 : 50) served as primary antibody. A biotinylated goat anti-rabbit immunoglobulin (Vector Laboratories, Burlingame, CA, USA; 1 : 400) was used as secondary antibody, followed by treatment with streptavidin-biotinylated horseradish peroxidase complex (Vectastain Elite ABC kit, Vector Laboratories). Using diaminobenzidine tetrahydrochloride (Sigma-Aldrich, Munich, Germany), sections were developed and counterstained with haematoxylin. Staining results for CAR were evaluated by estimating the percentage of epithelial cells showing specific immunoreactivity by an expert pathologist (MV) who was blinded for the clinical data. Immunopositivity at the plasma membrane and in the cytoplasm was assessed separately. The CAR status was classified as: negative (no immunoreactivity), weak (0–5% positive cells), moderate (5–50% positive cells), or strong (>50% positive cells). Only samples showing moderate or strong immunoreactivity were considered positive.

### Cell culture and generation of stably transfected cell lines

Human colon cancer cell lines SW480, SW620, DLD1, and HCT116 were obtained from the American Type Culture Collection (Rockville, MD, USA) and from the *‘Deutsche Sammlung von Mikroorganismen und Zellkulturen GmbH’* (Braunschweig, Germany), respectively, and cultured in the recommended growth media. Chinese hamster ovary cells were cultured in Ham's F12 containing 10% FCS. Colon cancer cell lines with functional CAR knocked down by specific CAR siRNAs or ectopic expression by human full-length CAR cDNA expressed under control of the CMV promoter in a pcDNA3.1 expression vector (‘hCARpcDNA3.1’ a kind gift of Dr J Bergelson), were generated as described previously (Anders *et al*, 2009b). Expression of CAR in pooled cell populations was determined by western blotting, and FACS was used for the assessment of CAR presence at the cell surface.

### Western blotting

Protein lysates were obtained as previously described (Anders *et al*, 2003a). Subsequently, equal amounts of protein lysates were loaded on reducing Laemmli gels, immunoblotted with specific antibodies against CAR (H-300: sc-15405, Santa Cruz Biotechnology), or *β*-actin (Sigma-Aldrich), and detected using the ECL system (Amersham Pharmacia, Piscataway, NJ, USA). Protein lysates of CHO and CHO-CAR cells were used as negative and positive controls, respectively.

### Measurement of CAR cell surface expression

Expression of CAR cell surface was analysed as previously described (Anders *et al*, 2003a). In brief, living cells were incubated with the anti-CAR antibody RmcB (a kind gift of Dr J Bergelson) in binding buffer (PBS containing 2% BSA and 10% normal goat serum) at 4°C for 45 min. Afterwards, cells were incubated with a Cy-3-conjugated anti-mouse antibody (Molecular Probes, Eugene, OR, USA). Analysis of stained cells was performed on a FACS-Calibur cytometer (Becton Dickinson, Franklin Lakes, NJ, USA).

### Assessment of colon cancer cell proliferation *in vitro* and *in vivo*

Cells were seeded onto six-well plates (*n*=3 × 10^5^ cells per well) in the recommended growth media containing 10% FCS. After 48 h, cells were detached using trypsin and counted using a haematocytometer (VWR International, Darmstadt, Germany). All experiments were performed in triplicate and repeated at least twice. To determine colon cancer cell growth *in vivo*, 10^7^ cells were injected subcutaneously into both flanks of 6- to 8-week-old male NOD/SCID mice obtained from The Jackson Laboratory (Bar Harbor, ME, USA). Tumour volumes were measured twice per week from day 8 onwards with a calliper in two dimensions and calculated according to volume=length × wide^2^/2. All animal experiments were performed according to German law at the ‘Experimental Pharmacology & Oncology GmbH (Berlin-Buch, Germany).

### Cell invasion into matrigel

Cells cultured in media with or without the anti-CAR antibody RmcB (a kind gift of Dr J Bergelson) were seeded onto the top of ‘BioCoat Matrigel Invasion Chambers’ (BD Biosciences, Bedford, MA, USA) containing 8-μm pore size PET membranes covered with matrigel matrix. Medium containing 10% FCS was added to the bottom well of the chambers as a chemoattractant, whereas serum-free medium was used as a control. Following 48 h at 37°C and 5% CO_2_, cells invaded the matrigel-coated membrane, located at the lower membrane surface, were fixed, and stained by crystal violet containing 10% ethanol. Cells of three representative areas in each well were counted at a magnification of × 100. Experiments were performed in triplicate and repeated at least twice.

### Colon cancer cell metastasis *in vivo*

The impact of CAR inhibition on the metastatic behaviour of colon cancer cells was assessed in 6-week-old female athymic SCID mice obtained from Charles River Laboratories (Sulzfeld, Germany). All mice received injections of 1 × 10^6^ cells (SW480 following CAR inhibition, SW620 with CAR upregulation or the respective controls) into the spleen. At 4 weeks, all mice were killed and spleen, liver, lung, and paraaortic lymph nodes were obtained, fixed in formalin, and embedded in paraffin. Subsequent histopathological analyses were undertaken using haematoxylin and eosin stained sections. All animal experiments were approved by the local governmental authorities (Landesamt für Gesundheit und Soziales, Berlin, Germany).

### Induction of apoptosis

To assess caspase activity, colon cancer cells were grown in 1 × 10^4^ 96-well culture plates. After 24 h of incubation, apoptosis was induced by addition of 50 ng ml^−1^ TRAIL (Biomol, Hamburg, Germany). Following another 48 h, caspase-3/7 activity was determined using Caspase-Glo 3/7 Assay Systems (Promega, Mannheim, Germany) at 1, 2, and 3 h after addition of Caspase-Glo 3/7 on a luminescence reader (Mithras LB 940, Berthold Technologies GmbH & Co. KG, Bad Wildbad, Germany). Hereafter, cell numbers were assessed photometrically following addition of crystal violet (Spectramax 340 PC Microplate Reader, Molecular Devices, Sunnyvale, CA, USA). All experiments were performed in triplicate and repeated at least twice.

### Statistical analysis

Statistical calculations were performed with the GraphPad Prism software (version 4.00; GraphPad Software, Inc., San Diego, CA, USA) using the *t*-test or *χ*^2^-test, when applicable.

## Results

### Differential abundance and subcellular distribution of CAR in colon tissue

**Figure 1:**
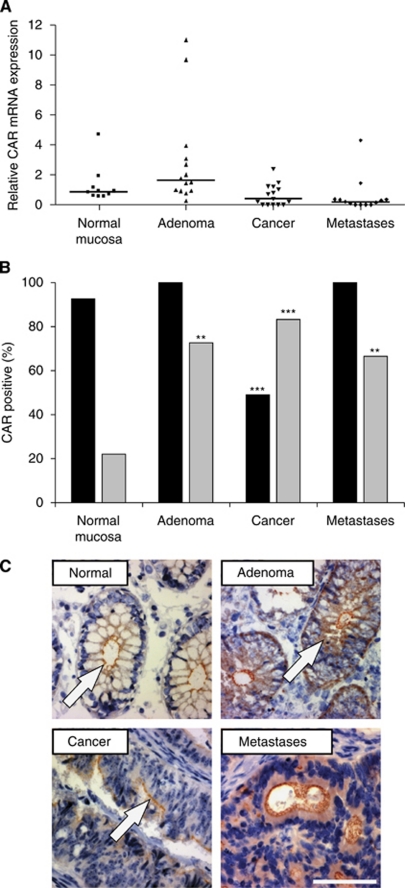
Presence and distribution of CAR in non-transformed colon mucosa, adenomas, primary, and metastatic colon cancers. Real time RT–PCR data represent median CAR mRNA expression level of non-transformed mucosa, adenomas, primary colon cancers, and metastases displaying highest CAR expression in adenomas (**A**). Immunohistochemistry revealed abundant CAR presence at the plasma membrane (black bars) in non-transformed mucosa samples, adenomas, primary colon cancer, and metastases. In colon cancers, a significant decrease of membranous CAR immunoreactivity was found. Cytoplasmic CAR immunopositivity (grey bars) was observed significantly more often in adenomas, primary colon cancers, and metastases (asterisks mark significant differences compared with normal mucosa) (**B**). Typical results for CAR immunostaining are shown in non-transformed colon mucosa, displaying CAR localisation at the apical plasma membrane (arrow), whereas in adenomas additional cytoplasmic CAR immunoreactivity was noted (arrow). CAR immunoreactivity in primary colon cancers was preferentially seen in the cytoplasm (arrow), whereas CAR presence at plasma membrane was frequently lost. In contrast, metastases displayed both membranous and cytoplasmic CAR presence (**C**) (magnification: × 100, bar=200 *μ*m).

We first studied the CAR mRNA expression by quantitative RT–PCR assays on randomly selected tissue specimens of normal colon mucosa (*n*=10), adenomas (*n*=14), primary colon cancers (*n*=15), and colon cancer metastases (*n*=13). These studies showed that the median Δ*C*_T_ values for CAR mRNA in comparison with normal mucosa (0.8805) increased significantly in adenomas (1.621; *P*=0.049), whereas a decrease of CAR mRNA expression was found in primary colon cancers (0.3920; n.s.) and colon cancer metastases (0.2040; *P*=0.032) (Figure 1A).

Using immunohistochemistry, we then analysed the presence and histoanatomical distribution of CAR. On a subcellular level, CAR was found at the plasma membrane of normal mucosa samples (93%), as well as adenomas and colon cancer metastases (100% each). Interestingly, membranous CAR immunoreactivity was found significantly less commonly in primary colon cancers (49%, *P*<0.0001). In contrast, the prevalence of cytoplasmic CAR immunopositivity was increased in adenomas (73%, *P*=0.0006), primary colon cancers (83%, *P*<0.0001), and metastases (67%, *P*=0.0019), compared with non-neoplastic mucosa (22%) (Figures 1B and C). For a subset of 11 patients, we were able to directly compare CAR presence in primary cancers and metastasis. Primary cancers were either entirely CAR negative (*n*=4 patients) or displayed a moderate CAR immunopositivity (mean=40% CAR-positive cells). In contrast, metastatic cancers of all patients were CAR immunopositive.

To assess whether CAR presence and/or subcellular distribution in colon cancer correlates with clinico-pathological parameters, we compared the CAR transcription (mRNA levels) and translation (immunoreactivity) with local tumour growth (T-category), nodal status (N-category), distant metastases (M-category), tumour grade (G), lymph (L-category) and blood-vessel-invasion (V-category), and the UICC tumour stage. None of these parameters correlated significantly with the expression and presence of CAR in primary colon cancers (data not shown).

### CAR expression in colon cancer cell lines

**Figure 2:**
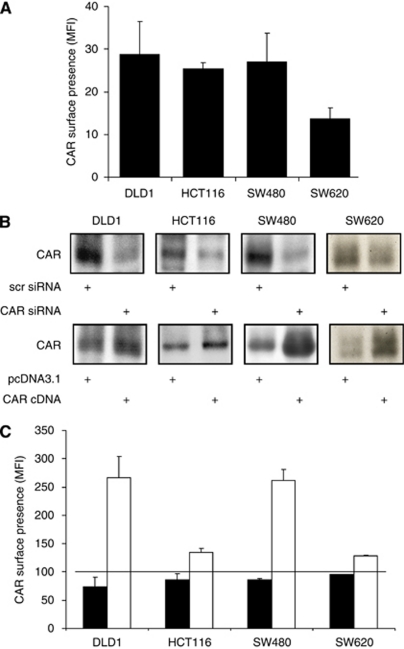
CAR expression and ectopic regulation in colon cancer cell lines. The presence of CAR at the surface was determined by FACS. Data represent the difference between mean fluorescence intensities (MFI) measurements for the anti-CAR antibody RmcB and a control containing the secondary antibody only (**A**). To regulate CAR expression in colon cancer cell lines, stable transfections with either a CAR-specific siRNA (upper panels) or a human full-length CAR expression vector ‘hCARpcDNA3.1’ (lower panels) and the respective controls (scrambled [scr] siRNA or pcDNA3.1) were performed. Differential CAR protein expression was assessed by western blotting (**B**) and CAR surface presence was determined by FACS (**C**).

In permanent colon carcinoma cell lines, FACS revealed the presence of CAR in all tested cell lines, with comparable CAR surface manifestation in DLD1, HCT116, and SW480, however, markedly lower levels in the SW620 cell line (Figure 2A). On the basis of these findings, we chose all cell lines for both CAR downregulation and forced expression. Western blotting confirmed reduced CAR protein expression in all cell lines after stable transfection of a CAR-specific siRNA and increased CAR protein levels following ectopic expression of hCARcDNA (Figure 2B). Presence of CAR at the surface in these cell lines was assessed by FACS in unfixed cells. Upon CAR inhibition, a moderate decrease was noted in the DLD1 cell line and, to a less extent, in HCT116 and SW480. In SW620 cells, no differences were noted. Following stable expression of CAR, full-length cDNA, a more than 2.5-fold increase in CAR surface presence was found in DlD1 and SW480 cells, whereas in HCT116 and SW620, a moderate increase was only found (Figure 2C).

### CAR inhibits growth of colon carcinoma cell lines *in vitro* and *in vivo*

**Figure 3:**
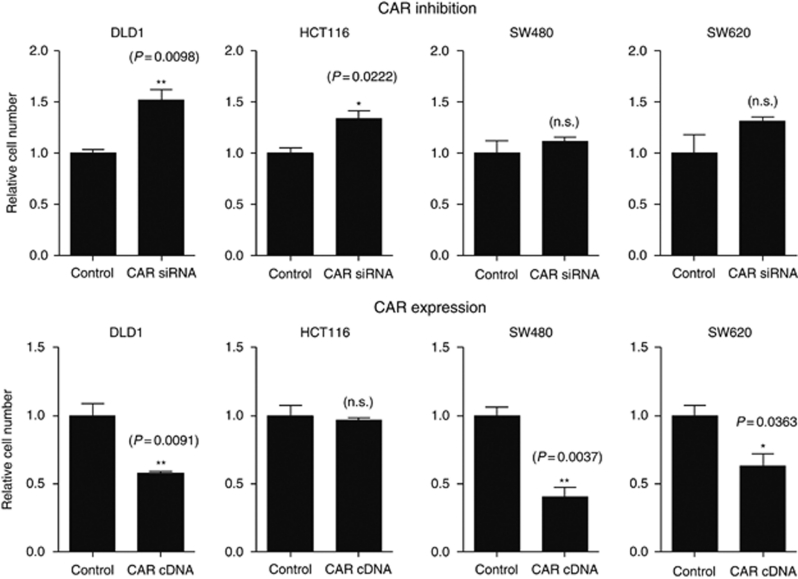
Influence of CAR on colon cancer cell proliferation. Proliferation was determined in DLD1, HCT116, SW480, and SW620 cells stably transfected with either a CAR-specific siRNA (upper panels) or a human full-length CAR expression vector ‘hCARpcDNA3.1’ (lower panels) and ‘vector only’ controls. Data represent typical results from a series of three independent experiments.

**Figure 4:**
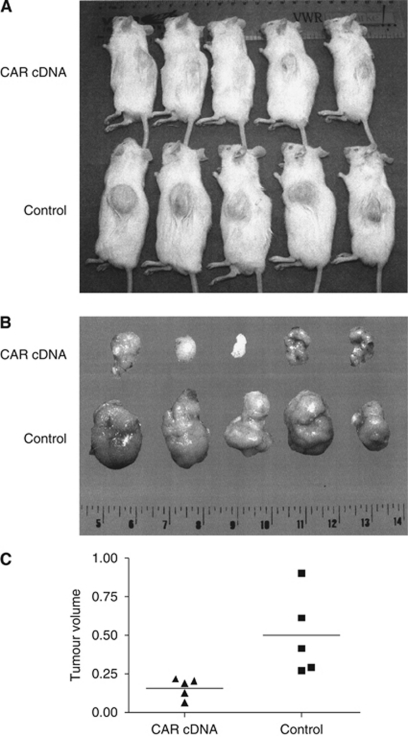
Impact of CAR on colon cancer growth *in vivo*. Ectopic CAR expression in SW480 cells resulted in a marked size reduction of xenograft tumours shown *in situ* (**A**) and freshly explanted (**B**). Determination of tumour volumes revealed a statistically significant difference between CAR overexpressing SW480 cells compared with the ‘vector only’ control cell line (**C**).

To clarify the influence of CAR on the growth of colon cancer cells, we performed *in vitro* proliferation assays following ectopic regulation of CAR expression. The RNAi-mediated functional CAR knockdown resulted in significantly higher cell numbers in DLD1 and HCT116 compared with vector controls, whereas for SW480 and SW620, a minor insignificant increase was found. Ectopic CAR upregulation resulted in a significant decline of cell numbers in SW480, SW620, and DLD1 compared with matching controls, whereas for the HCT116 cell line, no significant differences were noted (Figure 3). Subcutaneous xenograft tumours of colon cancer cell lines displayed a markedly reduced tumour size upon ectopic CAR upregulation compared with ‘vector only’ controls (SW480 (*P*=0.02) (Figure 4) and DLD1 (*P*=0.11) (data not shown)).

### CAR inhibition increases invasion of colon carcinoma cells

**Figure 5:**
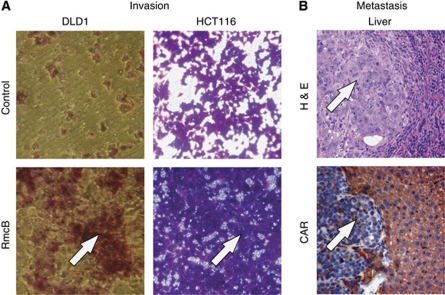
Inhibition of CAR promotes colon cancer cell invasiveness and metastasis. Blockade of the extracellular CAR portion using the anti-CAR antibody RmcB markedly increased the number of invading colon cancer cells *in vitro* (arrows) (**A**). RNAi-mediated knockdown of CAR in SW480 cells led to a significantly increased number of metastases (arrow) to the liver (lower panel demonstrates low CAR presence in metastatic cells (arrow) in contrast to adjacent CAR positive hepatic tissue) (**B**).

The impact of CAR on invasion of colon cancer cells following blockade of the extracellular portion of CAR was assessed using an *in vitro* assay. Incubation with the anti-CAR antibody RmcB, known to block CAR, markedly increased the invasiveness into matrigel of DlD1 and HCT116 cell lines compared with respective controls. However, SW480 and SW620 did not invade into matrigel neither with nor without RmcB (Figure 5A).

### Loss of CAR facilitates formation of liver metastases

The impact of CAR inhibition on the metastatic behaviour of colon cancer cells was assessed in 6-week-old female athymic SCID-mice. At 4 weeks after intrasplenic injection, all those animals displayed liver metastases, which had received SW480 following siRNA-mediated CAR knockdown. In contrast, liver metastases were found in only one animal (14%) injected with SW480 control cells. Moreover, the number of liver metastases in the group with SW480 siRNA cells was significantly higher (*P*=0.0169) (Figure 5B). In contrast, animals that had received injections of SW620 with upregulated CAR or controls did not display liver metastases (data not shown).

### CAR facilitates anti-apoptotic effects in colon cancer cells

The potential effect of CAR on the response of colon cancer cells to apoptotic stimuli was tested by treatment with TRAIL. Hereby, significantly higher caspase 3/7 levels were found in SW480, DlD1, and HCT116. In the SW620 cell line, a minor increase was noted only. Enforced CAR expression led to lower caspase 3/7 activity in DLD1 cells upon treatment with TRAIL, whereas no changes were found in HCT116 and SW620, and an increase in the SW480 cell line was found (data not shown).

## Discussion

The current study marks the first report of differential expression and subcellular distribution of CAR in the adenoma–carcinoma sequence of colon cancer. Our observations reveal an increase of CAR mRNA expression in adenomas and a downregulation in primary cancers and metastasis. On a subcellular level we noted (1) a significant increase of cytoplasmic CAR in neoplastic tissues compared with the normal mucosa, (2) reduced CAR presence at the plasma membrane in primary cancers, and (3) a ‘re-localisation’ of CAR to the plasma membrane in metastatic tumours. In conjunction with our data regarding the effects of CAR on growth, invasion, metastasis, and response to apoptotic stimuli, it may be speculated that CAR facilitates complex, stage-dependent functions in the course of colon carcinogenesis.

Our finding of high CAR mRNA expression and CAR protein localisation at the plasma membrane and within the cytoplasm in adenomas is in line with previous observations in early-stage breast cancer, Barrett's oesophagus, and early-stage oesophageal adenocarcinoma (Anders *et al*, 2003b; Anders *et al*, 2003b, 2009a). Therefore, it may be speculated that high CAR expression marks are a more general phenomenon in pre- and early malignant lesions. Functionally, CAR may promote early carcinogenesis as suggested for cervix and ovarian cancers, with CAR-expressing cell lines displaying less sensitivity towards apoptotic stimuli (Bruning *et al*, 2005). In line with this hypothesis, we found that colon cancer cell lines become more sensitive towards treatment with TRAIL following RNAi-mediated functional CAR knockdown. However, following CAR upregulation, either no change in caspase activity or contrary results were observed. Therefore, it remains unclear whether CAR contributes to the suppression of apoptosis in colonic neoplasias (Tsujitani *et al*, 1996).

Our observation of reduced CAR mRNA expression and CAR protein presence at the plasma membrane in primary colon cancers compared with non-transformed tissue is in agreement with reports for primary colon cancers and several other carcinomas (Heideman *et al*, 2001; Rauen *et al*, 2002; Sachs *et al*, 2002; Matsumoto *et al*, 2005; Korn *et al*, 2006; Buscarini *et al*, 2007; Zhang *et al*, 2008; Anders *et al*, 2009b). These findings are highly suggestive of an entity-independent downregulation of CAR in primary carcinomas. The loss of CAR at the plasma membrane may led to an impaired intercellular adhesion as prerequisite for cancer dissemination as suggested for ovarian, bladder, and gastric cancers as well as gliomas (Okegawa *et al*, 2001; Bruning and Runnebaum, 2004; Huang *et al*, 2005; Wang *et al*, 2005; Anders *et al*, 2009b). In accordance with this hypothesis, we found that blocking of the extracellular portion of CAR increases the invasiveness of colon cancer cells. Furthermore, we observed a marked rise in the metastatic behaviour of colon cancer cells following CAR knockdown. These findings are in line with a previous report showing reduced potential of murine CT26 cells to form lung metastases (Yamashita *et al*, 2007). In conjunction with our observations in human tissues, these data suggest that reduced CAR expression in primary colon cancers contributes to the spreading of colon cancer cells and therefore to disease progression.

Furthermore, our findings show that CAR is involved in the regulation of colon cancer cell proliferation. We noted reduced growth of colon cancer cells upon ectopic CAR regulation *in vitro* and *in vivo*. In line with these findings, specific CAR silencing led to complementary effects as shown *in vitro*. These results are in agreement with previous observations in gastric, oesophageal, bladder, and prostate cancer cell lines, as well as glioma cells (Okegawa *et al*, 2000, 2001; Huang *et al*, 2005; Anders *et al*, 2009a, 2009b), suggesting an entity-independent growth-suppressive function of CAR in human cancers. Underlying mechanisms, however, are not fully understood: Previously an accumulation of p21 and hypophosphorylated retinoblastoma protein upon CAR upregulation has been detected in human bladder cancer cell lines (Okegawa *et al*, 2001). On the basis of these findings, it has been hypothesised that cell-cell contact, initiated by membrane-bound CAR, may elicit a negative signal cascade modulating cell cycle regulators.

In colon cancer metastases, we noted abundant CAR immunopositivity confirming findings of a previous study describing ∼60% of colon cancer metastases in the liver to express CAR (Korn *et al*, 2006). Our findings are also in agreement with observations in local and distant metastases of prostate cancer, which present a strong membranous staining comparable to the non-transformed prostate mucosa, which is also significantly higher than the one in primary prostate cancers (Rauen *et al*, 2002). Moreover, these data are in line with findings for the cytoplasmic TJ protein ZO-1, a known binding partner of CAR (Kaihara *et al*, 2003). Our findings also do suggest that CAR presence at the cell surface of metastatic cancers results from differential distribution within the cell and not from elevated gene expression. Whether regulatory pathways such as Raf/MEK/ERK signalling that has been demonstrated to impact CAR localisation in colon cancer cells before are involved, remains to be elucidated (Anders, 2003a). The function of CAR on colon cancer metastases, however, remains hypothetical. Previously, CAR presence has been shown to correlate with the state of differentiation in metastatic colon cancer (Korn *et al*, 2006). As it has been shown that colorectal cancers undergo dedifferentiation at the primary site and ‘re-differentiate’ when metastastic to the liver (Brabletz *et al*, 2001; Kaihara *et al*, 2003). Reexpression of CAR may simply represent an epiphenomenon of cell differentiation. However, CAR may also be of functional importance as it might allow cancer cells to gain foothold at the metastatic site, as postulated for E-cadherin (De Marzo *et al*, 1999; Rubin *et al*, 2001; Liu *et al*, 2002). Recently, an isoform-specific localisation and regulation of CAR (CAR^Ex7^ and CAR^Ex8^) has been shown in human airway epithelia. Whether alternative splicing of the *CAR* gene occurs and impacts the development of colonic neoplasia, however, needs to be elucidated (Excoffon *et al*, 2010).

In conclusion, our results suggest that CAR mediates promoting as well as inhibiting functions in colon cancer biology, depending on the stage of the tumour development and progression. Interestingly, its inhibitory functions are in contrast to findings for another member of the TJ family, Claudin 1, which has been shown to promote cancer growth and metastatic properties (Dhawan *et al*, 2005). These findings underline the hypothesis of complex roles of TJ proteins in the process of colon cancer development.
